# The fucose salvage pathway inhibits invadopodia formation and extracellular matrix degradation in melanoma cells

**DOI:** 10.1371/journal.pone.0199128

**Published:** 2018-06-20

**Authors:** Tyler Keeley, Shengchen Lin, Daniel K. Lester, Eric K. Lau, Shengyu Yang

**Affiliations:** 1 Department of Cellular and Molecular Physiology, Penn State College of Medicine, Hershey, Pennsylvania, United States of America; 2 University of South Florida Cancer Biology Graduate Program, Tampa, Florida, United States of America; 3 Department of Tumor Biology, Comprehensive Melanoma Research Center, H. Lee Moffitt Cancer Center, Tampa, Florida, United States of America; University of Toronto, CANADA

## Abstract

The fucose salvage pathway is a two-step process in which mammalian cells transform L-fucose into GDP-L-fucose, a universal fucose donor used by fucosyltransferases to modify glycans. Emerging evidence indicates the fucose salvage pathway and the fucosylation of proteins are altered during melanoma progression and metastasis. However the underlying mechanisms are not completely understood. Here, we report that the fucose salvage pathway inhibits invadopodia formation and extracellular matrix degradation by promoting α-1,2 fucosylation. Chemically or genetically increasing the fucose salvage pathway decreases invadopodium numbers and inhibits the proteolytic activity of invadopodia in WM793 melanoma cells. Inhibiting fucosylation by depleting fucokinase abrogates L-fucose-mediated inhibition of invadopodia, suggesting dependence on the fucose salvage pathway. The inhibition of invadopodium formation by L-fucose or ectopically expressed FUK could be rescued by treatment with α-1,2, but not α-1,3/α-1,4 fucosidase, implicating an α-1,2 fucose linkage-dependent anti-metastatic effect. The expression of FUT1, an α-1,2 fucosyltransferase, is remarkably down-regulated during melanoma progression, and the ectopic expression of FUT1 is sufficient to inhibit invadopodium formation and ECM degradation. Our findings indicate that the fucose salvage pathway can inhibit invadopodium formation, and consequently, invasiveness in melanoma via α-1,2 fucosylation. Re-activation of this pathway in melanoma could be useful for preventing melanoma invasion and metastasis.

## Introduction

L-fucose is a 6-carbon sugar utilized by mammalian cells for glycosylation. Glycans can be modified by either core or branch fucosylation mediated by 13 different fucosyl transferases [1]. The deregulation of fucosylation in cancer has been reported to regulate cancer cell proliferation, adhesion, and motility [2, 3]. In melanoma, the core-fucosylation (α-1,6 fucosylation) mediated by fucosyltransferase 8 (FUT8) was reported to promote melanoma progression [4], while branched fucosylation through the α-1,2 linkage inhibits melanoma progression [4, 5]. Mammalian cells may use either de novo synthesis pathway or the salvage pathway to provide GDP-L-fucose for fucosylation [5]. It is recently reported that the expression of fucokinase (FUK), a key enzyme in the fucose salvage pathway, is remarkably down-regulated in metastatic melanoma, limiting GDP-L-fucose substrate availability [5]. Treating melanoma cells with L-fucose or overexpression of FUK resulted in decreased migratory potential as well as an increase in cell surface fucosylation. Administration of L-fucose through water supplementation not only slowed tumor growth, but also inhibited lung metastases in a melanoma mouse model [5], suggesting that the fucose salvage pathway can be targeted to suppress melanoma invasion and metastasis. However, it is not clear how protein fucosylation inhibits melanoma invasion and metastasis.

Increasing invasive capacity represents a crucial step in melanoma metastasis and progression [6, 7]. Breslow depth (level of invasion), which increases during melanoma progression from radial growth phase (RGP) to vertical growth phase (VGP) or regional/distant metastasis, correlates directly with worsening patient prognosis [8]. Interestingly, with RGP to VGP progression, the expression of FUK in melanoma is also dramatically downregulated, implicating a role for the fucose salvage pathway in the regulation of melanoma invasion [5]. Invadopodia are proteolytic membrane protrusions used by melanoma and other metastatic cancer cells to degrade extracellular matrix (ECM) and to facilitate metastatic dissemination [6, 9, 10]. Pro-invasion cues in the tumor microenvironment are able to induce the formation of invadopodia and degradation of ECM [11–14]. Many plasma membrane proteins, such as integrin receptors, growth factor receptors and transmembrane matrix metalloprotease, are modified by glycosylation. However, it is unclear whether or how protein glycosylation might affect invadopodium formation and ECM degradation.

In this study, we examined the role of the fucose salvage pathway in invadopodium formation and ECM degradation. Our data indicated that ectopic expression of FUK or L-fucose treatment in melanoma cells remarkably inhibited invadopodium assembly and ECM degradation. The inhibition of invadopodium assembly was mediated by the α-1,2, but not the α-1,3 or α-1,4 fucosylation of plasma membrane proteins. The ectopic expression of FUT1, a α-1,2 fucosyltransferase, was able to fully recapitulate the inhibition of invadopodium formation, confirming a role for the α-1,2 fucosylation in the regulation of invadopodia. Our data suggested that the fucose salvage pathway might inhibit melanoma progression by suppressing invadopodium formation and ECM remodeling.

## Methods and materials

### Cell culture

WM793 and WM245 melanoma cells were cultured in HyClone RPMI-1640 Media supplemented with 10% FBS and 1% Penicillin/Streptomycin. Cells treated with L-Fucose (Biosynth, F8060) were treated for 48 hours at 25μM or 50μM as indicated prior to experimentation.

### Gelatin coated coverslips

Glass cover slips (Fisherbrand 18Cir.-1, 12-545-100) were acid washed overnight in 1M HCl at 60°C. After thorough rinsing with diH_2_O, coverslips were washed with 1xPBS and stored in 20% EtOH. To coat, coverslips were removed from 20% EtOH and washed with 1xPBS. After last wash was removed, 100μL of 0.2% bovine gelatin (Sigma, G9391) in 0.2M NaHCO_3_ was applied to each coverslip, and allowed to stand for 20 minutes. After three washes of 1xPBS, 100μL of 0.5% Glutaraldehyde (Sigma, S7776) was applied to each coverslip and allowed to stand for 15 minutes. Coverslips were then washed extensively (10x) in 1xPBS to remove any residual glutaraldehyde. After final wash, coverslips were set gelatin side up into 12-well plates and either used immediately, or stored at 4°C for future use.

### Texas Red gelatin labeling and coating

The labeling and coating of Texas Red gelatin were performed was previously described [7, 15] with modifications. A 2% gelatin solution was made in 0.2M NaHCO_3_ pH 8.3. Texas Red-X Succinimidyl Ester (Invitrogen, T6134) was dissolved DMSO (Fisherbrand, BP231) at a concentration of 10mg/mL. 50μL of the reactive dye solution was mixed into 1.5mL of the filtered gelatin and allowed to incubate for 1 hour at room temperature with continuous stirring, protected from light. Unbound dye was then removed using a HiTrap desalting column. Recovered gelatin was diluted 1:8 and protected from bacterial growth with 2mM sodium azide (Sigma, S2002), and stored at 4°C. Acid washed coverslips (see above) were removed from 20% EtOH and washed three times with 1xPBS. After final wash, 100μL of poly-D-lysine (Sigma, P6403) was applied to each coverslip and allowed to stand for 20 minutes. Following another three washes in 1xPBS, 100μL of glutaraldehyde was applied to each coverslip and allowed to stand for 15 minutes. After three more washes of 1xPBS, 80μL of Texas Red labeled gelatin was applied to each coverslip and allowed to stand, protected from light, for 10 minutes. Excess gelatin was reclaimed and recycled. Coverslips were washed three times with 1xPBS, then quenched with 100μL of 5mg/mL sodium borohydride (Sigma, 452882) and allowed to sit for 15 minutes. Coverslips were then set into 12-well plates with gelatin side up, and washed three times with 1xPBS, then stored at 4°C protected from light, or used immediately.

### Proliferation assay

WM793 cells were plated into 12-well plates at equal densities in triplicate for each condition. After 4 hours, the media was changed on all plates, and one plate was washed once with 1xPBS and fixed with 10% buffered formalin as Day 0. At each 24-hour interval, another plate was washed and fixed. After plates were fixed for 5 days, cells were stained with crystal violet for 15 minutes. Plates were then washed in DI water until washes remained clear. The plates were then dried, inverted on paper towels overnight. Any residual water was aspirated. The cell bound stain was solubilized with 300μL 10% Glacial Acetic Acid per well, and placed on a shaker for 10 minutes. 50μL of each well was then transferred to a 96-well plate, and the absorbance read at 562nm.

### cDNA constructs

The pLenti-GFP, pLenti-GFP-hFUK, pLKO, and pLKO-hFUK-shB3 constructs were generously provided by Dr. Eric Lau. The CTRL shRNA (SHC016-1EA) and hFUKsh37856 (TRCN0000037856) were obtained from Sigma. Human FUT1 was subcloned from the Harvard Plasmid Repository (HsCD00345675) to pLenti-CMV-blast empty as follows: hFUT1 was amplified with BamHI and XhoI cutting sites, as well as a C-terminal flag tag using sense primer 5′-ATCAG GATCCCATGTGGCTCCGGAGCCATCGTC-3′ and antisense primer 5′-ACTCCTCGAGTCACTTGTCGTCATCGTCTTTGTAGTCAGGCTTAGCCAATGTCC-3’. The amplified product and the pLenti-CMV-blast vectors were digested using BamHI and XhoI and run on a 1% agarose gel. Digested vector and hFUT1 were purified using Wizard® Plus SV Gel and PCR Clean-Up System (A9282). The purified products were ligated for 2 hours at room temperature, and transformed to DH5α bacteria, and plated to ampicillin^+^ LB agarose plates. Multiple colonies were picked the following day, and amplified for Midi Prep DNA generation.

### Stable cell lines

HEK293T cells were used to package lentiviral particles as previously described [16]. Lentiviral vector cDNA (5μg) was combined with pSPAX (5μg), pMD2G (5μg) and polyethylenimine (Fisher, NC9197339) and added dropwise to 10cm plates of 60% confluent 293T cells. After 48 hours, virus was collected after each 14 hour interval for 4 days. Virus containing media was first centrifuged at 3000g for 15 minutes at 4°C to spin down cellular debris, then ultracentrifuged at 20,000rpm for 2 hours at 4°C to pellet virus. Pellets were suspended in growth medium and snap frozen in LN_2_, then stored at -80°C for future use. Virus was then used to infect WM793 cells, and after 48 hours, cells were subjected to antibiotic selection. Surviving cells were verified of complete selection by fluorescent microscopy for the hFUK constructs, or western blotting for the hFUT1 constructs.

qPCR: The qPCR primers for hFUK are as follows:

hFUK Fwd: 5’-CTGTATCCAGGCCAGTCACC-3’ Rev: 5’-CAGATTGTGCACTCCCAGGT-3’ GAPDH Fwd: 5’-TGAAGGTCGGAGTCAACGG-3’ Rev: 5’-AGAGTTAAAAGCAGCCCTGGTG-3’

WM793 cells were seeded at 5x10^5^ cells in 6cm plates and allowed to incubate overnight. The following day, plates were washed once with 1xPBS and total RNA was isolated using Qiagen RNeasy kit (Cat#74106). 2μg of total RNA was used for reverse transcription. cDNA was diluted 1:10 and used for qPCR with Power SYBR® Green PCR Master Mix (Applied Biosystems, 4367659).

### Invadopodia assay

WM793 cells were plated to 6cm dishes and allowed to grow for 48 hours. Parental WM793 cells were treated with 25μM Fucose or control water. Plates were washed once with 1xPBS and cells dissociated using enzyme free cell dissociation solution (Millipore, S-004-C). Cells were seeded to gelatin coated coverslips at 1x10^5^ cells per coverslip and allowed to incubate for 4 hours. Coverslips were then washed once with 1xPBS and fixed using fresh 4% paraformaldehyde (Sigma, 158127) for 20 minutes at room temperature. Cells were then stained with fluorescent phalloidin stains (Alexa Fluor phalloidin-488, -594, -647) based on the experimental conditions, and imaged for quantitation using wide-field microscopy. Samples were imaged using an Axiovert S100 upright microscope through a 63x/1.3 FLUAR Plan Apochromatic oil immersion objective. An attached Axiocam 503 mono charge-coupled camera and ZEN 2.3 blue edition (Zeiss) software were used to capture images. For representative images, samples were imaged using a Leica SMI8 inverted microscope, TCS SP8 confocal scanner, and a HC PL APO 63x/1.4 CS2 oil immersion objective. The 488, 552, and/or 638 STED lasers were used to excite the samples, and a tunable acousto-optical beam splitter was used to minimize crosstalk between fluorochromes. Sample emission was captured with Leica HyD hybrid detectors and images were prepared with the Leica Application Suite X.

To calculate percent degraded gelatin, ImageJ software (NIH) was used to determine total cell area in pixels. The suspected area of degradation was then duplicated, converted to 32-bit, and the threshold was set. Using the measure function, the percent area of selection that was degraded was calculated. The percent of the degradation of the gelatin under the entire cell was then calculated by multiplying the percent of degradation in the selected area by the total area of selection, then dividing by the entire cell area.

### Invadopodia precursor assay

WM793 cells were plated at 1x10^5^ cells per coverslip, and allowed to settle for 1 hour in complete RPMI. After 1 hour, cells were washed once with 1xPBS, and 1mL of 1% FBS RPMI (starvation media) was added to each well. The cells incubated for 18 hours, then FBS was added to the media to make up to 10% FBS for each well. Coverslips were fixed in 4% PFA every 15 minutes and images for quantitation and representation were acquired as mentioned.

### Fucosidase treatment

After cell dissociation using enzyme free solution, cells were washed three times in 1xPBS, then incubated in PBS with 0.08U/mL fucosidase (New England Biolabs, α-1,2:P0724S, α-1,3/4:P0769S) for 30 minutes at 37°C. Next, cells were washed three times in 1xPBS, then used in further experiments.

### Flow cytometery

Cells were harvested using enzyme free dissociation media, washed once with 1xPBS, and incubated with 1x PKH26 (Sigma, MINI26) for 1 minute at room temperature. Next, cells were washed three times in 1xPBS, then fixed in 2% paraformaldehyde in PBS for 45 minutes at room temperature and protected from light. Cells were then washed once with 1xPBS and blocked with 0.2% IgG- and protease-free BSA (Jackson ImmunoResearch, 001–000) for 30 minutes at room temperature. Cells were washed twice in 1xPBS and stained with 0.2μg/mL FITC-UEA-1 (Vector Labs, FL1061) for 1 hour at room temperature and protected from light. After two more washes with 1xPBS, cells were analyzed by flow cytometry. for levels of FITC-UEA-1 staining. FITC-UEA-1 staining levels were calculated as a ratio of the median UEA-1 values divided by median PKH26 values (where each condition was relative to the control). For flow cytometric experiments using GFP-EV- or GFP-FUK-expressing cells, the cells were harvested as above, divided in half for staining with PKH26 or with TRITC-UEA1 (EY Labs, R-2201-2) and analyzed by flow cytometry as described above.

### Western blotting

Cells were seeded to 6cm plates at 5x10^5^ cells per plate and allowed to incubate overnight. The following day, plates were washed once with ice cold 1xPBS, then lysed with a rubber policeman and ice cold RIPA buffer (150mM NaCl, 5mM EDTA, 50mM Tris pH 8.0, 1% NP-40) with 1mM phosphatase (ThermoScientific, 88667) and 1mM protease (Roche, 04693159001) inhibitor cocktails added. The following antibodies were used: FLAG (Sigma, F3165) at 1:50[17]00 dilution in 5% BSA (Fisher, BP1600) in TBST; GAPDH (Sigma, G8795) at 1:5000 dilution in 5% BSA in TBST.

### Bioinformatics

FUT1 and FUT2 mRNA expression data was retrieved from 5 previously published microarray datasets (GSE8401, GSE46517, GSE 7553, GSE15605 and GSE3189) [18–21]. The expression level data of FUT1 (probeset 206109_at) and FUT2 (probeset 208505_s_at) were ploted as scatted dot plot using Graphpad Prism 7.0 and the statistical analysis was performed using two-samples, two-tailed t-test.

### Matrigel invasion assay

Matrigel invasion by WM793 cells was performed using invasion Boyden chamber (8 μm) as we previously described previously [7, 15, 22, 23].

## Results

### L-fucose treatment inhibits invadopodia formation and extracellular matrix degradation

**Fig 1:**
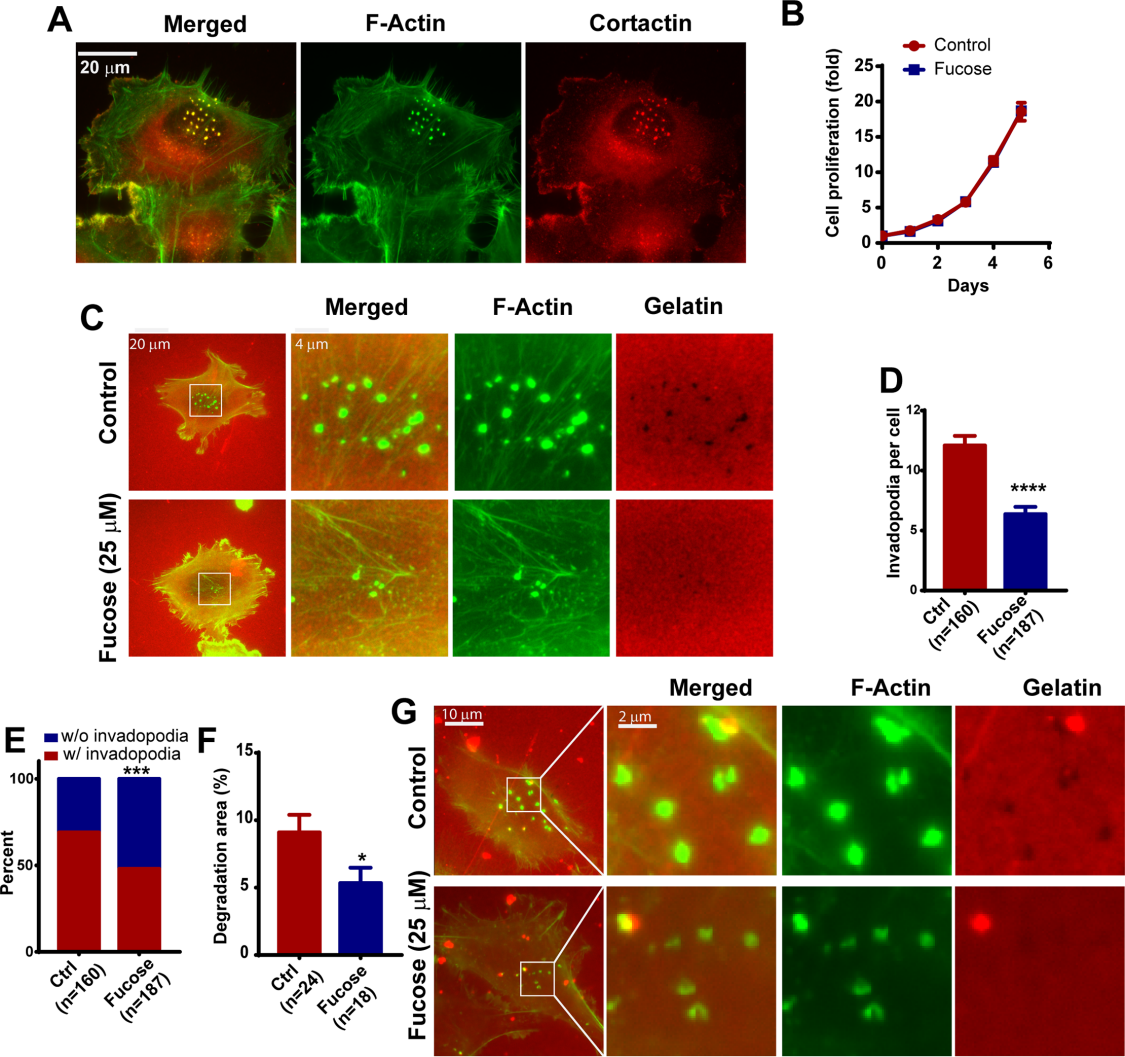
Fucose treatment inhibits invadopodia formation and ECM degradation. **A,** Representative images showing invadopodia in WM793 cells revealed by F-actin and cortactin double staining. **B**, the effect of fucose treatment on WM793 cell proliferation. **C,** representative invadopodia assay images showing the effects of 25μM L-fucose treatment on the formation of invadopodia and degradation of TexasRed labeled gelatin by WM793 cells. **D -F**, quantitation of the effect of fucose treatment on the average invadopodium number per cell (**D**), proportion of invadopodia-positive WM793 cells (**E**) and gelatin degradation area by WM793 cells (**F**). **G,** representative images showing the effects of fucose treatment on invadopodia formation and gelatin degradation in WM245 melanoma cells. *, *** and **** indicate *p<0*.*05*, *0*.*001 and 0*.*0001*, respectively. The p values were determined by two tailed, two sample t-test (in **D, F**) or two-tailed Fisher exact test (**D**). Numbers in parenthesis indicates the number of cells used in quantitation. Representative results from at least three independent replicates were presented.

Since FUK expression is suppressed in invasive and metastatic melanoma cells, and L-fucose supplementation inhibited melanoma metastasis, we examined the effects of L-fucose treatment on invadopodia formation. We have previously demonstrated that the WM793 melanoma cell line is an excellent model to study invadopodial regulation [7]. When plated on gelatin coated glass coverslips under normal growth condition, approximately 70% of WM793 cells were able to assemble arrays of robust invadopodia generally in areas underneath the nuclei [7]. These invadopodial puncta are cortactin and F-actin positive protrusions on the ventral site of the cell (Fig 1A), and are able to degrade fluorescent labeled gelatin [7]. L-fucose treatment had no effect on the proliferation of WM793 cells (Fig 1B). However, when pre-treated with 25 μM L-fucose for 48 hours, the average numbers of invadopodia per cell was decreased by about 50% when compared to vehicle control (dH_2_O)-treated WM793 cells (Fig 1C and 1D). The percentage of invadopodia-positive cells was also decreased from ~70% in control cells to ~40% in L-fucose treated cells (Fig 1E). To determine how the degradative capacity of invadopodia was affected by L-fucose, we further evaluated the effects of L-fucose-treatment on the degradation of fluorescence-labeled gelatin. The degradation of gelatin by invadopodia would leave black dots on a bright fluorescence background. Indeed there was a significant decrease in gelatin degradation in the L-fucose treated cells (Fig 1B and 1E.). The inhibition of gelatin degradation by L-fucose treatment was also observed in WM245, a melanoma cell line derived from radial growth phase melanoma (Fig 1F). Taken together, our data indicate that the number and degradative capability of invadopodia are inhibited by L-fucose treatment.

### Overexpression of FUK abrogates invadopodia formation and delays invadopodia initiation

**Fig 2:**
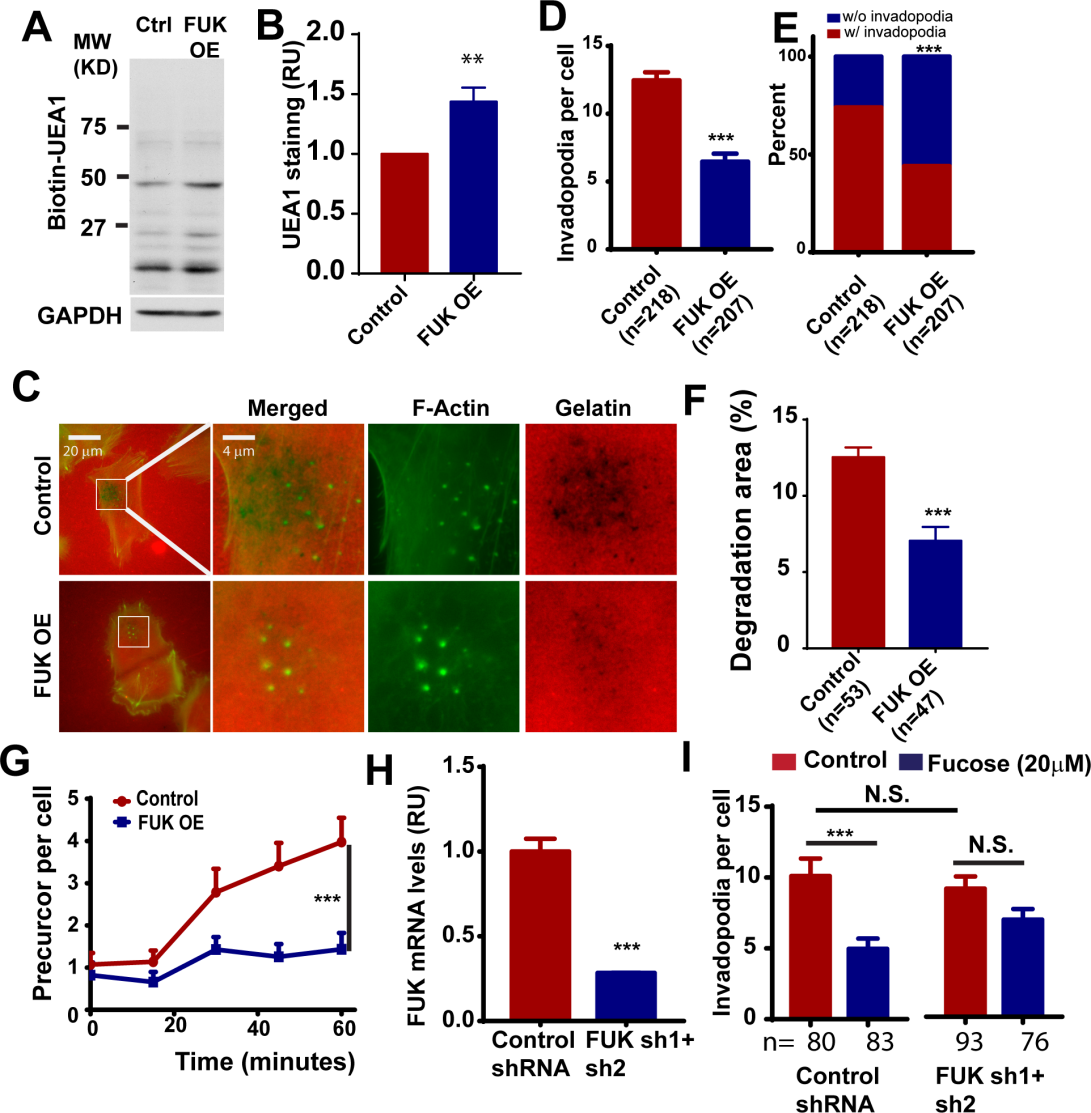
The ectopic expression of FUK in melanoma cells inhibits invadopodium formation and delays invadopodial initiation. **A,** the effect of FUK overexpression on the fucosylation of WM793 cell surface proteoglycans. The plasma membrane proteins from control and FUK OE WM793 cells were separated using SDS-PAGE and detected by biotin-UEA1. **B,** the effect of FUK overexpression on cell surface fucosylation as detected by UEA1 staining and flow cytometry. **C**, representative images showing the effects of ectopically expressed FUK on invadopodium formation and TexasRed gelatin degradation in WM793. **D and E,** quantitation of the effect of ectopically expressed FUK on the invadopodium number per cell (**D**) and the proportion of invadopodia positive cells (**E**) in WM793. **F,** quantitation of the effects of FUK overexpression on gelatin degradation. **G,** the effects of ectopic FUK on the initiation of the assembly of invadopodia induced by 10% FBS. **H,** qPCR assay showing the effects of FUK sh1+sh2 on the mRNA transcript levels of FUK in WM793 cells. **I,** quantitation of the effects of FUK knockdown on fucose-mediated inhibition of invadopodium formation in WM793 cells. *** indicates *p<0*.*001*, as determined by two-tailed, two sample t-test (**D, G, H, I**) or two-tailed Fisher’s exact test (**E**). Numbers in parentheses indicates the number of cells used in quantitation. Representative results from at least three independent replicates were presented.

To determine whether activation of the fucose salvage pathway is sufficient to inhibit invadopodium formation, we ectopically express FUK in WM793 cells. The expression of FUK was verified by qPCR (data not shown). The expression of FUK increased the fucosylation of cell surface proteoglycans (Fig 2A and 2B). The control and FUK OE cells were seeded to gelatin coated coverslips and the formation of invadopodia was determined by phalloidin staining. Similar to L-Fucose treatment, we found that the ectopic expression of FUK resulted in about 50% drop in average invadopodia per cell (Fig 2C and 2D). Additionally, the proportion of invadopodia positive cells was reduced by about 40% in FUK OE group (Fig 2E). We also found that the overexpression of FUK attenuate the degradative ability of invadopodia in WM793 cells (Fig 2F). Taken together, our data indicate that augmenting the fucose salvage pathway by ectopically expression FUK is sufficient to inhibit invadopodia formation and ECM degradation.

To understand the mechanism by which the fucose salvage pathway regulates invadopodia, we determined the effects of ectopic FUK expression on invadopodial initiation. WM793 cells were starved in 1% FBS overnight to reduce the basal invadopodial assembly. The cells were then stimulated with 10% FBS and the extents of FBS-induced invadopodial formation were quantified at indicated time points over one hour (Fig 2G). We found that FBS stimulation remarkably induced invadopodial formation in control cells, as we previously reported [7]. In contrast, FBS-induced invadopodium initiation was significantly inhibited in WM793 cells expressing ectopic FUK (Fig 2G), suggesting that the fucose salvage pathway in melanoma regulates invadopodial initiation.

### FUK is required for L-fucose-mediated inhibition of invadopodium formation

As the phosphorylation of L-fucose by FUK is a crucial first step in the generation of GDP-L-fucose for fucosylation, we next investigated whether FUK is required for L-fucose-mediated inhibition of invadopodium formation. By combining 2 shRNA targeting hFUK, we were able to reduce the mRNA transcript levels of FUK in WM793 by about 70% (Fig 2H). To determine whether FUK is required for fucose-mediated inhibition of invadopodium formation, control or FUK knockdown WM793 cells were incubated for 48 hours with 20 μM L-fucose, and then the effect of fucose treatment on invadopodium formation were evaluated by phalloidin staining. As shown in Fig 2H, FUK knockdown itself has no significant effect on invadopodium formation. However, the depletion of FUK significantly reduced the L-fucose-mediated inhibition (Fig 2I). Taken together, our data suggested that FUK is essential for L-fucose-mediated inhibition of invadopodium assembly.

### α-1,2 fucosylation is responsible for the inhibition of invadopodia formation by fucose

**Fig 3:**
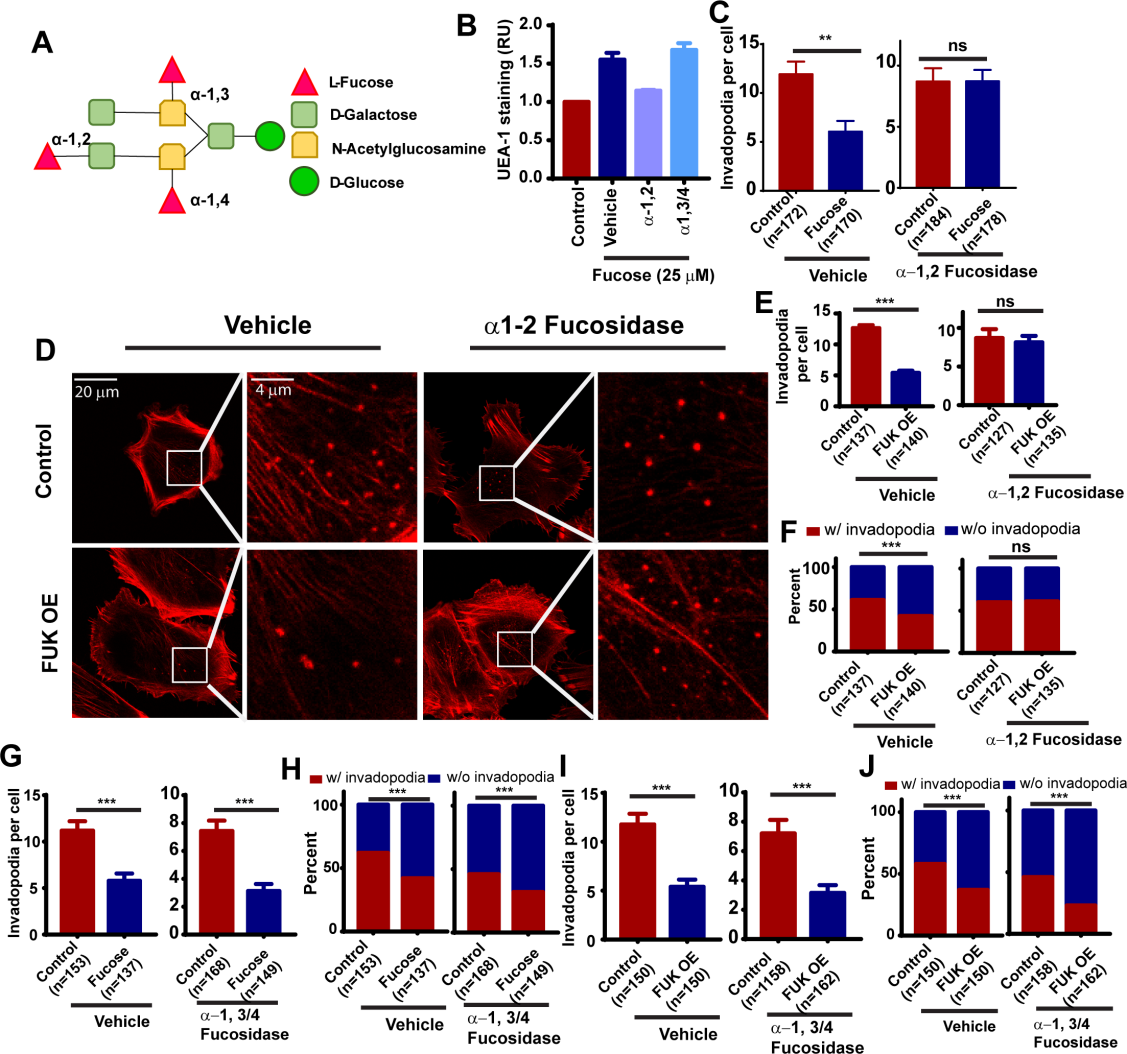
The fucose salvage pathway inhibits invadopodia through α1–2 fucosylation. **A,** schematic illustration showing the modification of glycans by α-1,2, α-1,3 and α-1,4 branched fucosylations. **B,** the effects of L-fucose and fucosidase treatment on cell surface α1–2 fucosylation, as determined by UEA-1 staining and flow cytometry. **C,** quantification of the effects of α-1,2 fucosidase treatment on fucose-mediated inhibition of invadopodium formation in WM793 cells. **D,** representative invadopodium assay images showing the effect of α-1,2 fucosidase treatment on FUK-mediated inhibition of invadopodium formation in WM793 cells. **E and F,** quantitation of the effect of α-1,2 fucosidase treatment on the invadopodium number per cell (**E**) and the proportion of invadopodia positive cells (**F**) in control or FUK OE WM793. **G and H,** quantitation of the effect of α-1,3/4 fucosidase treatment on the average invadopodium number per cell (**G**) and proportion of invadopodia positive cells (**H**) in control or fucose-treated WM793. **I and J,** quantitation of the effect of α-1,3/4 fucosidase treatment on the average invadopodium number per cell (**I**) and proportion of invadopodia positive cells (**J**) in control or FUK-overexpressed WM793. ** and *** indicates *p<0*.*01 and 0*.*001*, respectively, as determined by two-tailed, two sample t-test (**C, E**) or two-tailed Fisher’s exact test (**F, G**). ns, not statistically significant. Numbers in parentheses indicates the number of cells used in quantitation. Representative results from at least three independent replicates were presented.

We noted that the L-fucose- and FUK-mediated inhibition of invadopodia was abrogated when cells were lifted with trypsin instead of by protease-free dissociation methods (data not shown). We reasoned that the neutralization of fucosylation effects by trypsin digestion might be due to proteolysis of fucosylated transmembrane proteins. To determine whether plasma membrane protein fucosylation is mediating the inhibition of invadopodium formation, we used fucosidase to remove the fucosylation of cell surface proteoglycans. Proteoglycan can be modified by α-1,2, α-1,3 or α-1,4 branched fucosylations (Fig 3A). Control or L-fucose-treated WM793 cells were lifted from the plates using protease-free dissociation buffer and incubated with fucosidase to remove branched fucosylation, and the effects of fucosidase treatments on cell-surface α-1,2 fucosylation were evaluated by UEA-1 lectin staining. As shown in Fig 3B, L-fucose treatment increased cell surface UEA-1 staining, suggesting an increase in α-1,2 L-fucose linkages. The increase in UEA-1 staining was abrogated by treatment with α-1,2 fucosidase (but not with α-1,3/α-1,4 fucosidase), confirming the specificity of fucosidase treatment and UEA-1 staining (Fig 3B). Interestingly treatment with α-1,2 fucosidase sufficed to completely abrogate the inhibition of invadopodia by L-fucose (Fig 3C). To determine whether FUK-mediated inhibition of invadopodia was dependent on α-1,2 fucosylation, we treated control of FUK OE WM793 cells with α-1,2 fucosidase. The treatment with α-1,2 fucosidase partially rescued the inhibition of invadopodia formation by FUK (Fig 3D–3F). The removal of α-1,2 fucosylation by fucosidase also restored the proportion of invadopodia-positive cells in FUK OE group. In contrast, the treatment with α-1,3/α-1,4 fucosidase did not rescue invadopodia formation in fucose-treated or FUK-overexpressed WM793 cells (Fig 3G–3J), suggesting that the inhibition of invadopodia by the fucose salvage pathway is mediated by cell surface α-1,2 fucosylation but not by the α-1,3/α-1,4 fucosylation.

**Fig 4:**
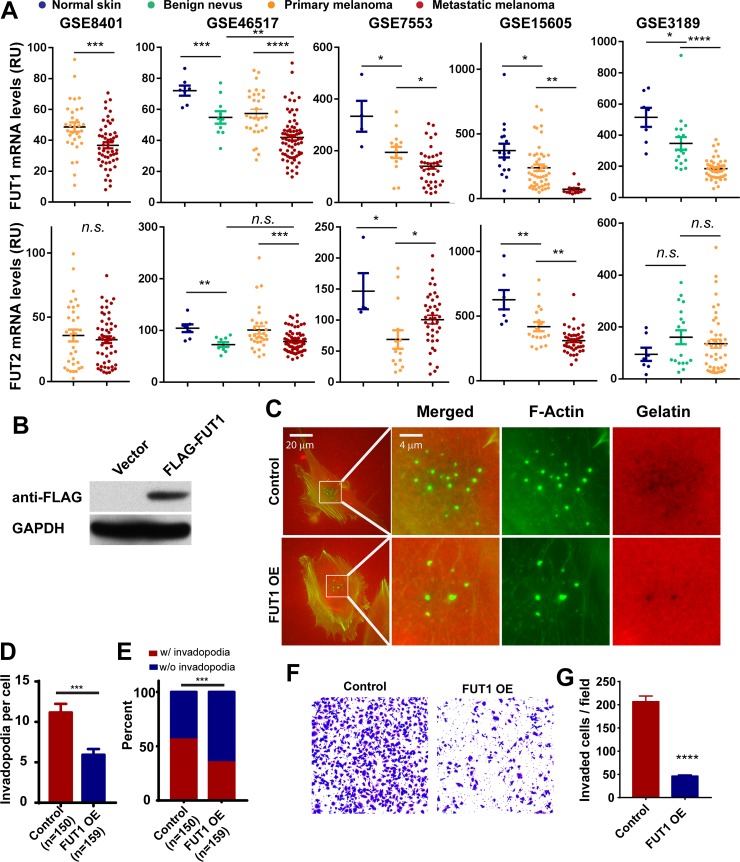
The ectopic expression of α-1,2 fucosyltransferase FUT1 inhibits invadopodia in WM793 cells. **A,** comparing the expression levels of FUT1 and FUT2 mRNA in normal skin, benign nevi, primary melanoma and metastatic melanoma in five microarray datasets. **B,** the ectopic expression of Flag-FUT1, as determined using anti-FLAG Western blotting. **C,** representative invadopodia assay images showing the effect of ectopically expressed FUT1 on invadopodium formation and gelatin degradation in WM793 cells **D and E,** quantitation of the effect of ectopically expressed FUT1 on the invadopodium number per cell (**D**) and the proportion of invadopodia positive cells (**E**) WM793. **F and G,** representative images (F) and quantitation data showing the effects of FUT1 overexpression in WM793 cells on Matrigel invasion. *, *** and **** indicates *p< 0*.*05*, *0*.*001 and 0*.*0001*, as determined by two-tailed, two sample t-test (**A, D, G**) or two-tailed Fisher’s exact test (**E**). Numbers in parentheses indicates the number of cells used in quantitation. Representative results from at least three independent replicates were presented.

Fucosyltransferase 1 and 2 (FUT1 and FUT2) are the fucosyltransferases responsible for the transfer of L-fucose to glycans via α-1,2 linkages and has been shown to be involved in inhibition of melanoma cell migration and adhesion [19]. We examined the changes of FUT1and FUT2 expression levels during melanoma progression in five previously published datasets (GSE8401, GSE46517, GSE 7553, GSE15605 and GSE3189) [18–21]. As shown in Fig 4A, FUT1 mRNA levels were downregulated in malignant melanoma when compared to benign nevi or normal skin from human patients (Fig 4A). The levels of FUT1 were further suppressed in metastatic melanoma when compared to primary melanoma (Fig 4A). In contrast, there was no clear correlation between FUT2 mRNA levels and melanoma progression in these datasets. Although in some datasets there appeared to be down-regulation of FUT2 mRNA in metastatic melanoma when compared to primary melanonma (GSE46517,GSE15605), in other datasets there was either no changes or increases in FUT2 expression with melanoma progression (GSE8401, GSE7553, GSE3189). Therefore, to further evaluate whether α-1,2 fucosylation is sufficient to inhibit invadopodia, we ectopically expressed Flag-tagged FUT1 in WM793 cells (Fig 4B). As shown in Fig 4C and 4D, ectopic expression of FUT1 resulted in approximately 50% reduction in the average number of invadopodia when compared to the vector control cells (Fig 4D). Additionally, analysis of the percentage of cells that presented invadopodia showed an approximate 40% reduction in the cells overexpressing FUT1 as compared to the empty vector control cells (Fig 4E). In line with the previous experiments, FUT1 overexpression also inhibited the Matrigel invasion activity of WM793 (Fig 4F and 4G), suggesting that α-1,2 fucosylation mediated by FUT1 is sufficient to inhibit invadopodium formation and melanoma invasion.

## Discussion

The differential deregulation of fucosylation has been correlated with tumorigenesis and tumor progression in various cancers [5, 24–27]. More recently, a systemic glycomics study suggested that the role of glycosylation in melanoma progression could be linkage dependent [4].

The activity of FUK in catalyzing the crucial phosphorylation of L-fucose in the salvage pathway is reportedly attributable for up to 40% of total cellular fucosylation [28]. In metastatic melanoma, although the expression of FUK is suppressed by the PKCε-ATF2 pathway, the ectopic expression of FUK or treatment with L-fucose significantly inhibits melanoma cell migration/invasion in vitro and lung metastasis in mouse models. These data indicate that the FUK-mediated salvage pathway represents an important determinant of invasive/metastatic capacity and therapeutically actionable target for suppressing melanoma.

However, the mechanisms by which FUK and the fucose salvage pathway regulates melanoma progression are not completely understood. Here, we present evidence that the ectopic expression of FUK and L-fucose treatment inhibit the formation and the proteolytic activities of invadopodia, suggesting that the fucose salvage pathway might control melanoma invasion through invadopodium-mediated ECM remodeling. It is interesting to note that the depletion of FUK in WM793 melanoma cells at least partially abrogated the L-fucose-mediated inhibition of invadopodium formation, suggesting that the downregulation of FUK in metastatic melanoma might desensitize melanoma cells to the anti-metastatic effects of L-fucose. FUK overexpression and fucose treatment similarly inhibit invadopodia in melanoma cells. However, in some experiments it appeared that fucose treatment may decrease invadopodial size (Fig 1C and 1G), while FUK overexpression may result in larger invadopodia. The underlying mechanism and potential significance for such difference remained to be determined.

Core-fucosylation (α-1,6 fucosylation) catalyzed by FUT8 is highly elevated in metastatic melanoma, and promotes melanoma progression and metastasis by regulating L1CAM cleavage and L1CAM-mediated invasion [4]. In contrast, α-1,2 branched fucosylation of glycans attenuates melanoma growth and metastasis [4, 5], suggesting that the modification of glycans by different fucosyltransferases might have drastically different functional consequences. Treatment of melanoma cells with α-1,2 fucosidase but not α-1,3/α-1,4 fucosidase sufficedto abrogate the inhibition of invadopodia by L-fucose and FUK, suggesting the inhibitory effects of the fucose salvage pathway is mediated through the α-1,2 fucosylation of plasma membrane glycans. The ectopic expression of FUT1, a α-1,2 fucosyltransferase, sufficed to recapitulate the inhibition of invadopodium assembly and ECM degradation, further supporting this notion. It is interesting to note that the expression of FUT1 and FUT2, the two α-1,2 fucosyltransferases, are suppressed during melanoma progression [4], and the levels of α-1,2 fucosylation are inversely correlated with the survival of melanoma patients [5]. Although all fucosyltransferases use GDP-L-fucose as fucosylation donors, it is possible that the fucose salvage pathway might preferentially provide GDP-L-fucose for certain fucose transferases through compartmentalization or spatial-temporal regulation.

The assembly and maturation of invadopodia are controlled by many glycosylated plasma membrane proteins such as integrins, growth factor receptors, ion channels/exchangers, matrix metalloproteases, etc. Our findings that the modulation of FUK and the fucose salvage pathway inhibits both the initiation of invadopodial assembly and the proteolytic activity of invadopodia establishes a foundation for future exploration of how the fucose salvage pathway regulates the functions of such crucial invadopodial proteins, and importantly, metastatic capacity in melanoma.
